# The Inflammation-Energy Metabolism Axis: A Central Driver of Sarcopenia-Osteoporosis: A Narrative Review

**DOI:** 10.1007/s00223-025-01473-8

**Published:** 2026-01-07

**Authors:** Yifan Jiang, Xiaonan Qi, Haijian Cui, Yingtao Huang, Yibo Lv, Yu Yang, Xiaosheng Yao, Dongxiang Yang

**Affiliations:** 1 First Clinical School, Liaoning University of Traditional Chinese Medicine, Shenyang, 110847 China; 2 Orthopedics and Traumatology, Affiliated Hospital of Liaoning University of Traditional Chinese Medicine, Shenyang, 110847 China; 3 Graduate School, Liaoning University of Traditional Chinese Medicine, Shenyang, 110847 China; 4 Liaoning University of Traditional Chinese Medicine, Shenyang, 110847 China

**Keywords:** Sarcopenia-osteoporosis, Inflammaging, Energy metabolism, Muscle-bone crosstalk, NF-κB signaling

## Abstract

Sarcopenia-osteoporosis, the concomitant loss of muscle and bone mass, is a geriatric comorbidity that significantly increases the risk of falls, fractures, and mortality. Despite the acknowledgment of muscle-bone crosstalk, the underlying mechanism driving their simultaneous decline remains poorly understood, resulting in a lack of effective integrated therapies. This review aims to elucidate the “inflammation-energy metabolism axis” as the central driver of this condition. This review contends that sarcopenia-osteoporosis is not a coincidental comorbidity but a single pathological syndrome driven by the failure of the inflammation-energy metabolism axis. Its pathophysiology is a self-sustaining vicious cycle where chronic low-grade inflammation (inflammaging) and dysregulated energy metabolism (notably mitochondrial dysfunction and insulin resistance) reciprocally amplify each other, leading to synchronous muscle and bone deterioration. The review first details how chronic inflammation, via pathways like NF-κB and the NLRP3 inflammasome, promotes muscle catabolism and bone resorption. Second, it explains how an energy crisis, stemming from mitochondrial damage and insulin resistance, impairs anabolic processes in both tissues. Finally, the underlying cycle is revealed: inflammation disrupts metabolic pathways (e.g., PI3K/AKT/mTOR), while metabolic stress releases DAMPs that further fuel inflammation, trapping the muscle-bone unit in a catabolic state. Understanding this central axis necessitates a paradigm shift from single-target treatments to systemic interventions. Future strategies should focus on disrupting this vicious cycle through combination therapies (e.g., anti-inflammatory and pro-metabolic agents), gut microbiota modulation, and exercise, offering novel approaches to this debilitating comorbidity.

**Supplementary Information:**

The online version contains supplementary material available at 10.1007/s00223-025-01473-8.

## Introduction

### Sarcopenia-Osteoporosis Comorbidity: A New Clinical Issue

Sarcopenia-osteoporosis refers to a comorbid condition of loss of skeletal muscle mass and function with reduced bone mineral density. It was first proposed by Neil Binkley in the year 2009 [1]and significantly increases the risk of falls, fractures, and death during old age [2]. Studies have shown that sarcopenic women are 12.9 times more probable to develop osteoporosis, 2.7 times more probable to fracture, and 2.1 times more probable to fall within 12 months compared to non-sarcopenic women [3]. The epidemiology of sarcopenia-osteoporosis worldwide was reported on a meta-analysis and systematic review to be 18.5% in the population aged 46.6 to 93 years [2]. In the United Kingdom, approximately £4 billion annually is spent treating osteoporosis-related fragility fractures [4]; like osteoporosis, sarcopenia puts health systems in billions of dollars every year globally [5]. Because of the cost and public health impact, better treatments are being investigated for. Currently, it is believed that no uniform intervention programs and single guidelines for sarcopenia-osteoporosis are in place [6]. While osteoporosis has a number of pharmacological therapies (e.g., bone density-enhancing drugs), sarcopenia treatment is predominantly non-pharmacological. Where various mechanisms can explain the effect of anti-osteoporotic therapy on sarcopenia [7], no article has yet provided a in-depth analysis of the comorbid mechanism driven by inflammation-mediated energy metabolism in muscle-bone interaction. This review aims to fill this critical gap by proposing that the ‘inflammation-energy metabolism axis’ functions as the central driver of this comorbidity, offering an integrated framework that explains the simultaneous deterioration of both tissues.

### Muscle-Bone Interaction: from Mechanical Coupling To an Endocrine Network

Looking back at previous viewpoints and information, Julius Wolff and others had the perception that mechanical load may affect the structure of the skeleton of organisms [8]. More current studies indicate that bone and skeletal muscle interact in a state of homeostasis through mechanical coupling; upon attaining a threshold level of force from skeletal muscle to bone, bone resorption converts to bone formation [9].

Mechanical loading due to contraction of skeletal muscles (e.g., tensile stress, fluid shear stress) is imposed on the skeleton, and its bone cells sense these mechanical stresses through structures such as the cytoskeleton, primary cilia, integrins, and ion channels (e.g., Piezo1) [10]. The Wnt pathway is most prominent in inducing transduction of cell biology pathways in osteocytes [11]. The increased mechanical stress by muscle contraction inhibits sclerostin and Dkks, which is possibly a principal factor accountable for bone loss in pathophysiological disorders, thereby eliminating the inhibition of the Wnt/β-catenin signaling pathway, promoting osteoblast differentiation and bone formation, as well as skeletal muscle regeneration [12]. This is evidence that muscle-to-bone mechanical signals can be transmitted to biochemical molecular signals.

There have been some studies in recent times that proved the physiological regulation of the skeletal and musculoskeletal systems by various different regulatory mechanisms. They are under upstream endocrine regulation by central and peripheral organs, and there exists a complex web of autocrine/paracrine signaling interactions between the musculoskeletal system and bone metabolism. Upstream endocrine regulators are largely made up of GH-IGF-1 axis hormones, sex hormones, adipokines (such as leptin, adiponectin, visfatin) [13], IL-6, and vitamin D [14]. Autocrine/paracrine regulators include skeletal muscle-secreted myokines and bone-secreted osteokines.

### The Inflammation-Energy Metabolism Axis as the Central Driver of Comorbidity

Dysregulated energy metabolism is a state characterized by the disruption or inappropriate activation of prevailing pathways of energy production, conversion, or use in the body, leading to the compromised functions of cells or tissues in meeting the energy homeostasis required for physiological processes. This is manifested primarily as mitochondrial dysfunction and core pathway dysfunctions such as glycolysis, oxidative phosphorylation, fatty acid metabolism, and amino acid metabolism, resulting in disordered glucose metabolism, deranged lipid metabolism, and a lipotoxic environment [15].

Chronic low-grade inflammation is a pathophysiological state characterized by an inflammatory process at a subclinical level, without overt symptoms such as pain, fever, fatigue, or any other functional change [16]. Inflammation triggers the systemic release of reactive oxygen species (ROS) and pro-inflammatory cytokines (such as tumor necrosis factor TNF-α, interleukins), which react with and have a cascade effect [17]. It triggers reduced insulin signaling, hypoxia, oxidative stress, cellular senescence, endoplasmic reticulum stress, mitochondrial damage, and the NF-κB pathway activation [18]. In pathogenesis of sarcopenia-osteoporosis, chronic low-grade inflammation and dysregulated energy metabolism are not distinct entities but an integrated system termed as the “inflammation-energy metabolism axis.” It is an age- or diet-metabolic-overload-induced feedback-regulated and self-amplified system that is the pivotal generator of sarcopenia-osteoporosis comorbidity.

## The Muscle-Bone Unit: A Tightly Coupled Endocrine System

The answer to the microscopic mechanistic connection between bone and skeletal muscle is, as stated above, “muscle-bone crosstalk.” Because bone and myocytes are invaded by an extensive, very vascularized system, molecules may be transported into the systemic circulation with the assistance of the extracellular fluid of the endomysium as a vehicle so that myokines may diffuse through to the periosteum and exert their actions [19].

### Bone Regulation by Myokines

Irisin is an exercise-induced and muscle-secreted proteolytic product of fibronectin type III domain-containing protein 5 (FNDC5) [20]. Irisin has been shown to be capable of inducing the proliferation and differentiation of osteoblasts directly, enhancing bone-forming ability by activating pathways such as bone morphogenetic protein (BMP) [21]. Conversely, low concentrations of Irisin have been shown to induce mouse bone marrow progenitor cell differentiation into osteoclasts, suggesting it may also act as a counter-regulatory hormone in bone resorption and highlighting its complex role in bone remodeling [22]. In models of osteoporosis in ovariectomized mice and hindlimb-suspended mice, Irisin was found to inhibit bone loss, increase bone microarchitecture (e.g., trabecular thickness and number), and increase bone strength and bone mineral density (BMD) [23, 24].

IL-6 is a member of the IL family and can be secreted by several cells such as fibroblasts, monocytes, macrophages, T cells, endothelial cells, adipocytes, and myocytes [25]. IL-6 exerts a dual regulation on bone formation. It activates markers of osteoblast differentiation through the conventional pathway [26]. During an inflammatory state, overexpression of IL-6 inhibits periosteal bone formation through the trans-signaling pathway [27] and indirectly increases osteoclast activity by activating RANKL expression, leading to the dominance of bone resorption over bone formation [28].

IGF-1 is an insulin family of polypeptide hormones that functions through binding to the high-affinity tyrosine kinase receptor (IGF-1R) on the cell surface. It promotes osteoblast differentiation and bone matrix mineralization through paracrine action and can inhibit osteoclast function, thereby bone resorption [29]. In an in vitro experiment on bone marrow mesenchymal stem cells (BMSCs), IGF-1 significantly enhanced the proliferation and osteogenic differentiation of cells through activation of the Wnt/β-catenin pathway [30].

FGF-21 is a fibroblast growth factor (FGF) family member and cell signaling protein that is mainly produced by macrophages. Experiments proved that the effect of FGF-21 on bone cells (bone mesenchymal stem cells, osteoblasts, osteoclasts) is a double-edged sword. In a Duchenne muscular dystrophy (DMD) mouse model, FGF-21 exerts a direct positive influence on RANKL-induced osteoclastogenesis, which causes increased bone resorption. FGF-21 also affected BMSCs to inhibit bone formation by suppressing osteogenic differentiation and promoting adipogenesis [31]. However, in an ischemic stroke model, the upregulation of FGF-21 was shown to prevent BMSCs from apoptosis via the PI3K/AKT pathway to preserve bone regeneration in patients with bone defects [32].

### Osteokine Control of Skeletal Muscle

Osteocalcin (OC) is an osteokine secreted by osteoblasts. Mouse models of OC deficiency in animal experiments had a high degree of reduction of skeletal muscle mass and myofiber integrity that could be reversed by exogenous supplementation with OC [33]. It was also able to reconstitute the exercise endurance of old mice (15 months) to that of young mice (3 months) [34]. In a human clinical trial, a population of middle-aged and older adults showed that levels of OC correlated with muscle mass, which was mediated by insulin sensitivity [35].

PGE-2 is being released in high levels from osteocytes [36]. By activating its receptor EP4, it promotes the proliferation and expansion of muscle stem cells (MuSCs), thereby significantly augmenting the capacity for muscle regeneration. Acute PGE-2 administration enhances muscle repair and the rate of recovery of muscle strength, while NSAID-induced inhibition of PGE-2 hinders muscle regeneration [37]. The reduction of bone-derived PGE-2 was found to be linked to atrophy of fast-twitch fibers. The reduction of osteocyte PGE-2 secretion in a transgenic mouse model resulted in a marked reduction in fast-twitch muscle content and inhibited myotube formation [38]. Wnt signaling participates in the regulation of the myogenic program and MuSC differentiation [39]. Wnt3a, a key signaling molecule of the Wnt pathway, enhances the contractile force of skeletal muscle and supports myoblast differentiation through the activation of the Wnt/β-catenin pathway [40].

### Muscle-Bone Unit Homeostasis Depends on the Integrity of the Signaling Network

**Fig. 1:**
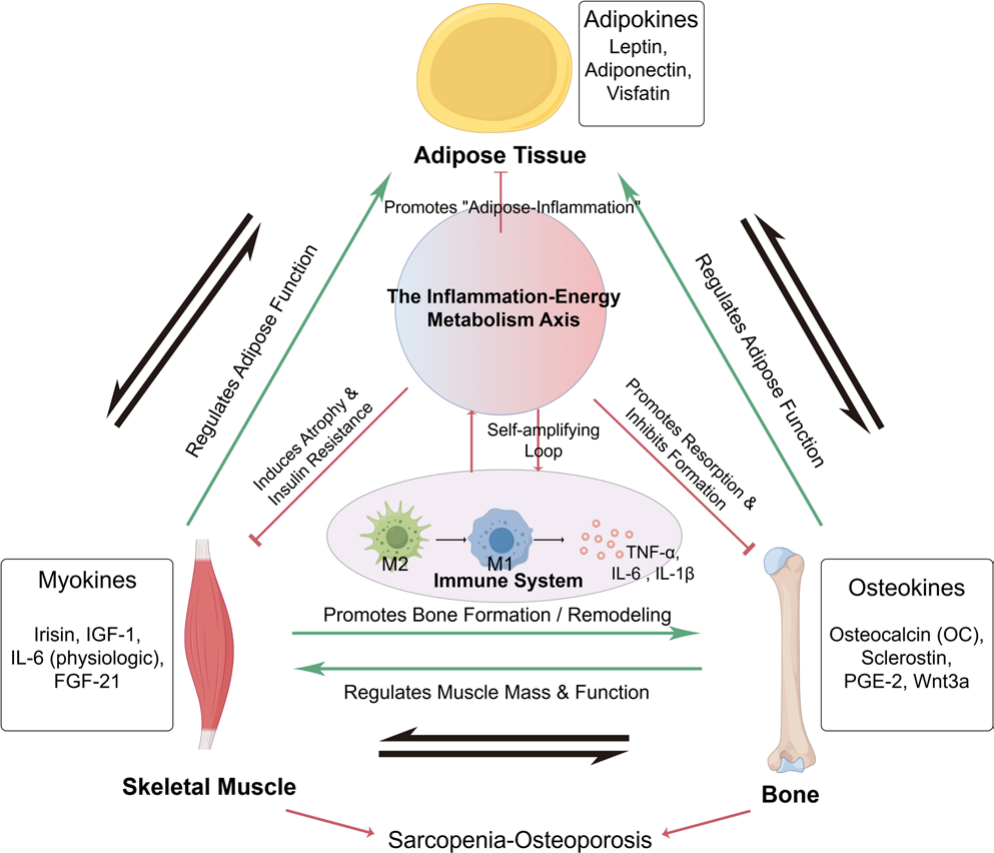
A Systemic view of inter-organ crosstalk in musculoskeletal health and disease. Musculoskeletal integrity relies on a dynamic and reciprocal communication network involving skeletal muscle, bone, adipose tissue, and the immune system. In a state of health, this network operates in a tightly regulated feedback loop, illustrated here by the green arrows. Muscle contraction stimulates the release of myokines like irisin and IGF-1, which in turn support bone formation. Bone reciprocates by secreting osteokines, such as osteocalcin and PGE-2, to enhance muscle function and regenerative capacity. This muscle-bone unit is further integrated into a larger systemic dialogue with adipose tissue, whose adipokines help govern whole-body energy balance. The central “Inflammation-Energy Metabolism Axis” represents the critical node where this homeostatic balance can unravel. When this axis is compromised, as depicted by the red arrows, the physiological dialogue shifts to a pathological cascade. This disruption promotes a state of chronic inflammation within adipose tissue, induces insulin resistance in muscle, and skews bone remodeling towards net loss. Ultimately, this systemic failure entraps the musculoskeletal system in a feed-forward loop of decline, giving rise to the concurrent pathologies of sarcopenia and osteoporosis

The skeletal muscle and bone systems regulate each other via endocrine signals. These components form a network that controls bone and muscle homeostasis. This crosstalk involves not only mechanical coupling of skeletal muscle with bone but also biochemical signal transmission. Bone and skeletal muscle both serve as endocrine organs that control one another. If the signaling network is deregulated, muscle mass and bone mass significantly diminish. Moreover, this imbalance can disrupt the exchange of stem cells between bone and muscle, further deteriorating the regenerative power of both systems [41]. This complex crosstalk extends beyond the muscle-bone unit, forming a systemic endocrine network that also involves adipose tissue and the immune system, all of which are critical for musculoskeletal homeostasis (Fig. 1).

## The First Driver: Chronic Low-Grade Inflammation

### Inflammaging: the Shared Ground between Sarcopenia-Osteoporosis

Sarcopenia and osteoporosis are both closely associated with the presence of inflammation. More and more evidence indicates that among the multiple factors controlling skeletal muscle homeostasis and ultimately leading to sarcopenia, inflammation is one of the most pivotal [42], and different studies also indicate that chronic low-grade inflammation is one of the primary causes of osteoporosis [43]. As age advances, the body develops a sterile, low-grade chronic inflammatory state without apparent infection, known as “inflammaging.” It has been associated with the development of numerous diseases and is considered the common basis for sarcopenia-osteoporosis comorbidity [44]. Such inflammation directly impacts the muscle protein balance of breakdown and synthesis by the continuous release of pro-inflammatory cytokines (such as IL-6, TNF-α), accelerating loss of muscle mass and function [45] and systemically increasing bone resorption. Clinical evidence shows circulating levels of the routine inflammatory marker CRP are related to bone mineral density [46]. More novel immuno-hematological indicators such as the Systemic Immune-Inflammation Index (SII) and the Platelet-to-Lymphocyte Ratio (PLR) were also identified to be closely associated with sarcopenia severity and risk of postmenopausal osteoporosis, and this reflects the body’s chronic low-grade inflammation level [47, 48].

### Core Pro-inflammatory Pathways Destruction of Muscle-Bone Unit

The NF-κB Pathway: A Master Switch for Muscle Atrophy and Bone Resorption.

NF-κB signaling pathway activation plays a profound role in musculoskeletal homeostasis. In muscle, the ubiquitin-proteasome system is a crucial system for muscle protein hydrolysis, of which Atrogin-1 and MuRF-1 are two most important E3 ubiquitin ligases, and the expression of these two factors is closely related to skeletal muscle atrophy [49]. Previous studies have identified that overexpression of IKKβ (an important NF-κB activator) increases the upregulation of MuRF1 levels and leads to skeletal muscle atrophy [50]. NF-κB also induces inflammation-related molecules (e.g., TNF-α, IL-1β, IL-6) to increase expression, directly or indirectly causing muscle atrophy, and can also inhibit the myogenic differentiation process by disturbing my-factors and impairing satellite cell function [51]. Within the skeletal system, NF-κB signaling pathway plays a similarly critical destructive role. It can influence osteoblast differentiation and function by regulating BMP/Runx2 and Wnt-β-catenin pathways [52]. NF-κB under inflammatory conditions can indirectly drive bone resorption and cause bone loss by initiating osteoclast differentiation signals (such as RANKL/RANK/OPG) via classical and non-classical pathways [53]. Therefore, the NF-κB pathway acts as a “master switch,” and its activation over time leads to simultaneously muscle catabolism and bone resorption.

NLRP3 Inflammasome: A Connector Between Cellular Stress and Systemic Inflammation.

Inflammasome is a multi-molecular complex whose activation could lead to the maturation of IL-1β and IL-18 and induce pyroptosis via the caspase-1 pathway. Of these, the NLRP3 inflammasome is well characterized, and multiple upstream mechanisms have the capacity to activate it, including ROS, mitochondrial damage, lysosomal disruption, ATP-mediated P2 × 7 receptor activation, efflux of potassium, and calcium influx [54]. Upon aging, stimulation of the NLRP3 inflammasome stimulates chronic low-grade inflammation [55], and the accumulation of ROS-damaged and dysfunctional mitochondria in skeletal muscle, in turn, stimulates the formation of the NLRP3 inflammasome [56]. Upon activation, it on the one hand activates osteoblast pyroptosis and inhibits their differentiation [57]. On the other hand, the released inflammatory cytokines such as IL-1β and IL-18 affect skeletal muscle and bone. IL-1β, by binding to IL-1R, can stimulate the NF-κB pathway by a MyD88-dependent mechanism, triggering ubiquitin-proteasome system-mediated protein degradation and leading to decreased muscle mass, thus producing sarcopenia-osteoporosis systemically.

### Double-Hit Effect of Key Inflammatory Mediators (IL-6, TNF-α)

In the complex network of inflammaging, IL-6 and TNF-α are key mediators that cause a “double-hit” on the muscle-bone unit. IL-6 has a complex regulatory mechanism in the development of sarcopenia-osteoporosis. IL-6 stimulates bone formation by activating osteoblast differentiation markers through the canonical pathway [26] and, in the context of inflammation, inhibits periosteal bone formation through the trans-signaling pathway [27] and indirectly stimulates osteoclast activity by raising RANKL expression and aiding in bone resorption more than bone formation [28]. In muscle, low levels of IL-6 induce proliferation and differentiation of muscle satellite cells by activating JAK/STAT3, growth and regeneration of the muscle; high levels of IL-6 suppress muscle growth and lead to muscle atrophy [58]. The activity of TNF-α is more clearly degradatory. In muscle, it causes muscle atrophy and myofiber abnormality through the activation of NF-κB signaling and inhibition of MyoD protein stability [59], but low concentrations have the potential to trigger myogenesis via the p38 MAPK pathway [60]. In bone metabolism, TNF-α tends to “facilitate bone resorption and inhibit bone formation.” It inhibits the proliferation and differentiation of osteoblasts [61] and enhances osteoclast activity by upregulating RANKL expression [62].

## The Second Driver: Dysregulated Energy Metabolism

### Bioenergetic Needs of the Muscle-Bone Unit

Energy metabolism is a physiological process that includes various molecules, cells, and processes at the systemic level and can be defined as glycolysis, oxidative phosphorylation, lipid metabolism, and amino acid metabolism [15]. Skeletal muscle and bone are high-energy-requiring tissues, and their homeostasis depends on an incessant and adequate energy supply. Dysregulation of energy metabolism, i.e., disturbance or derangement of the major pathways of energy production, conversion, or use in the body, will lead to failure of the cells or tissues to sustain the energy homeostasis required for normal physiological function [15], and hence inevitably at the cost of the structure and function of these two tissues.

### Mitochondrial Dysfunction: the Cell’s “Energy Crisis”

As the most important energy-producing organelle of eukaryotic cells, mitochondria play an irreplaceable role in maintaining cellular energy homeostasis. Mitochondrial dysfunction is a central promoter of cellular oxidative stress [63]. Stressful state leads to the generation of chemicals like ROS and malondialdehyde (MDA), which are able to activate various transcription factors like NF-κB, AP1, and STAT [64]and cause the body’s chronic low-grade inflammatory state. Thereafter, inflammation may itself inhibit mitochondrial biogenesis (e.g., by reducing PGC-1α expression), heightening oxidative stress and energy crisis [65]. Moreover, dysfunctional mitochondria may release mitochondrial DNA (mtDNA) into the cytoplasm, activating the NLRP3 inflammasome and spreading the inflammatory reaction [66]. This dysfunction ultimately leads to an insufficient supply of ATP, which cannot meet the demand of muscle protein synthesis and osteoblast function, and directly inhibits the proliferation and differentiation potential of muscle satellite cells [67] and reduces the function of osteoblasts [68] via ROS.

### Insulin Resistance (IR) and Lipotoxicity

Metabolic syndrome (MetS), a mixture of abdominal obesity, dyslipidemia, hypertension, and disrupted glucose metabolism, is highly linked with IR [69]. There is a bidirectional regulation between IR and inflammation: chronic low-grade inflammation interferes with the insulin signaling pathway by inflammatory cytokines, leading to decreased peripheral tissue insulin sensitivity [70]. The hyperglycemia and hyper-free fatty acids induced by IR, in turn, can further augment oxidative stress and inflammatory responses, forming a vicious circle [71]. Sarcopenic obesity is a most effective risk factor for MetS. Its deposition of adipose tissue or hypertrophy of adipocytes can lead to adipose tissue inflammation. This lipid deposit within muscle cells and other organs leads to lipotoxicity. This environment is characterized mainly by reduced concentrations of IL-10 and IGF-1, and increased concentrations of GDF-8, leptin, IL-1, IL-6, and TNF-α, thereby increasing inflammation in fat and muscle [72] and increasing osteoclast activation by enhancing RANKL expression in osteoblasts, aggravating osteoporosis [73].

### Dysregulation of Core Energy-Sensing Pathways

#### The PI3K/AKT/mTOR Pathway: the “Brake” on Anabolism

PI3K/AKT pathway is a signal transduction cascade consisting of phosphoinositide 3-kinase (PI3K) and protein kinase B (AKT). PI3K can phosphorylate PIP₂ into the second messenger PIP₃, causing AKT translocation from the cytoplasm to the cell membrane and activating downstream target proteins such as mTOR [74, 75]. In a state of persistent low-grade inflammation, increased concentrations of inflammatory cytokines such as TNF-α and IL-6 will inhibit the degree of phosphorylation of AKT, downregulating the activity of the PI3K/AKT/mTOR pathway while upregulating the FoxO3a pathway, ultimately decreasing muscle protein synthesis [76]. This state also inhibits the activity of IGF-1 [77], but IGF-1 can promote gly-gogen metabolism and mitochondrial function by the PI3K/AKT/FOXO1 pathway [78], and enhance the mitochondrial function of osteoblasts in resisting apoptosis that is induced by pro-inflammatory factors [79].

#### The AMPK Pathway: the “Failure” of the Energy Sensor

AMPK is a heterotrimeric complex responsible for regulating energy status by sensing changes in the intracellular AMP/ATP ratio [80]. When activated, AMPK stimulates fatty acid oxidation and glucose uptake and possesses anti-inflammatory properties. On the one hand, it inhibits inflammatory signaling such as NF-κB via activation of SIRT1 [81]. On the other hand, it triggers anti-inflammatory M2 macrophage polarization via repolarization of cellular metabolism (e.g., enhancing oxidative phosphorylation) [82]. In the context of aging and metabolic disorders, the activity and responsiveness of AMPK are decreased, leading to its “failure” function. In experiments, scientists discovered that ablation of AMPKα1 enhances mesenchymal stem cell (MSC) senescence, reducing their osteogenic differentiation capabilities. At the same time, the SASP-induced senescence is also responsible for secreting inflammatory mediators that inhibit muscle regeneration [83].

## The Core Argument: the Vicious Cycle of Inflammation and Metabolic Dysfunction

### How Inflammation Drives Dysregulated Energy Metabolism

Chronic inflammation is a very strong inducer of dysregulated energy metabolism. For example, chronic levels of pro-inflammatory cytokines TNF-α and IL-6 can decrease the phosphorylation state of AKT through various mechanisms, inhibiting the activity of the key anabolic pathway PI3K/AKT/mTOR [76]. In muscle, this directly results in faulty protein synthesis. At the same time, inflammation also amplifies ROS generation by activating pathways such as NF-κB and upregulating the activity of NADPH oxidase [84]. Overwhelming ROS directly result in causing mitochondrial DNA and respiratory chain damage, leading to mitochondrial dysfunction and energy crisis for ATP synthesis [65]. Furthermore, the inflammatory environment can induce metabolic reprogramming of immune cells (e.g., macrophages) to transition from an anti-inflammatory M2 phenotype dependent on oxidative phosphorylation to a pro-inflammatory M1 phenotype dependent on glycolysis. This transition releases more inflammatory cytokines such as IL-1β and TNF-α, reinforcing and amplifying the inflammatory response [85, 86].

### How Dysregulated Energy Metabolism Fuels Inflammation

Dysregulated energy metabolism is also “fuel” for inflammation. Damaged mitochondria can release mtDNA into the cytosol. As a damage-associated molecular pattern (DAMP), it directly activates the NLRP3 inflammasome, promoting the maturation and release of IL-1β and IL-18 and triggering a strong inflammatory response [56, 66]. At the same time, the lipotoxic environment activates insulin resistance and increased levels of free fatty acids promotes the polarization of adipose tissue macrophages to produce cytokines that promote IR and muscle wasting [87]. This adipose tissue inflammation due to metabolic dysbalance leads to local and systemic chronic low-grade inflammation.

### A Point of Convergence: the Dual Regulation of the Wnt/β-catenin Pathway

**Fig. 2:**
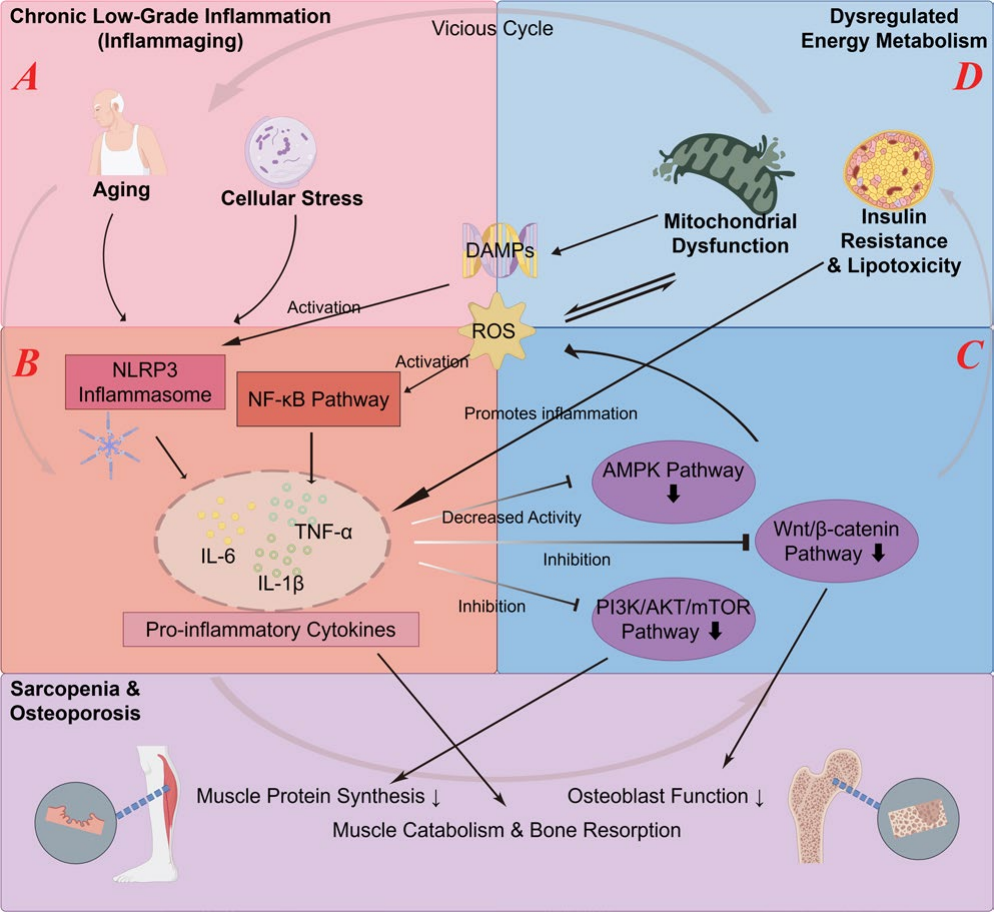
The vicious cycle between inflammation and energy metabolism driving sarcopenia-osteoporosis. This schematic details the core pathogenic engine proposed to drive sarcopenia-osteoporosis: a self-perpetuating cycle between chronic inflammation and metabolic failure, and use A, B, C, D to label the different stages of this vicious cycle. **A**.The starting point of the cycle: The cascade is often set in motion by aging and cellular stress, which trigger a low-grade inflammatory state.** B**.Key inflammatory hub mediators: Inflammatory hub, including the NF-κB pathway and the NLRP3 inflammasome, become chronically active, unleashing a barrage of cytokines like TNF-α and IL-6. These mediators directly assault the musculoskeletal system, tipping the balance towards muscle catabolism and bone resorption.** C**.Key metabolic dysregulation pathways: Crucially, this inflammatory environment sabotages the cell’s metabolic machinery. It suppresses vital energy-sensing and growth pathways, such as AMPK and PI3K/AKT/mTOR, and the resulting oxidative stress (ROS) cripples mitochondrial function. This leads not only to an energy deficit but also to systemic insulin resistance.** D**.Vicious cycle: The cycle then closes and intensifies as metabolic dysfunction fuels the fire of inflammation. Damaged mitochondria, for example, release DAMPs, which act as potent triggers for the very same inflammatory pathways that initiated the damage. This feedback loop creates a sustained, destructive state that systematically dismantles both muscle and bone, explaining their synchronous degeneration in older age

The Wnt signaling pathway is at the core of muscle-bone homeostasis and is a perfect case study for demonstrating the coordination of inflammatory and metabolic signals. For one, the Wnt signaling pathway is among the top biological pathways that allow osteocyte transduction [11]. The mechanical stress from contraction of the skeletal muscle can de-repress the Wnt/β-catenin signaling pathway by inhibiting the expression of sclerostin and Dkks and thereby promote osteoblast differentiation and bone formation [12]. At the same time, Wnt signaling is also involved in the regulation of the myogenic program and differentiation of the muscle stem cells; for example, Wnt3a can promote the differentiation of myoblasts by activating the Wnt/β-catenin pathway [39, 40]. However, in the pathological state of sarcopenia-osteoporosis, inflammatory signals (e.g., NF-κB) can inhibit the Wnt-β-catenin pathway and osteoblast activity [52, 88]. Thus, mechanical, inflammatory, and metabolic signals converge here, all determining together the fate of the muscle-bone axis. This intricate and self-perpetuating vicious cycle, which forms the central argument of this review, is schematically illustrated in Fig. 2.

## Clinical Translation and Future Outlook

### Potential Strategies Targeting the “Inflammation-Energy Metabolism Axis”

Based on the basic model of the “inflammation-energy metabolism vicious cycle” founded in this review, the pattern of intervention for sarcopenia-osteoporosis should immediately change from the traditional single-target thinking to the more macroscopic and systemic multi-target regulation mode. This model easily explains the lack of effectiveness in clinical practice of single-target treatments, i.e., anti-resorptive agents (e.g., bisphosphonates) or simple nutritional supplements (e.g., vitamin D and calcium)—they only target the downstream aspects of the disease and fail to break the upstream vicious cycle driving muscle atrophy and bone loss [89]. Therefore, future therapeutic interventions need to be tailored to address inflammatory and metabolic pathways in parallel to develop a synergistic effect that addresses both the symptom and the root cause [90].

One highly promising strategy is to explore the concurrent application of “anti-inflammatory” and “pro-metabolic” therapies. With regard to anti-inflammatory therapies, Senolytics therapy (e.g., dasatinib and quercetin combination), which is directed against senescent cells, is highly promising. Senescent cells are an important source of pro-inflammatory factors (SASP), and removing these cells can directly impair the foundation of “inflammaging,” ameliorating age-related tissue dysfunction, including sarcopenia and osteoporosis [91]. Additionally, drugs against key inflammatory mediators, such as IL-6 receptor antagonists (tocilizumab), can also protect the muscle-bone unit by blocking inflammatory signals directly. With regard to pro-metabolic interventions, AMPK activator metformin is not just a conventional anti-diabetic drug but also possesses many anti-inflammatory and anti-aging effects via activation of the AMPK pathway, such as inhibition of NF-κB and improvement of mitochondrial function, and therefore has been a focus of attention for delaying age-related diseases [92]. The other is supplementing with NAD + precursors (e.g., NMN or NR) to boost intracellular NAD + levels, activate deacetylases like SIRT1, and thereby improve mitochondrial function, suppress inflammation, and overall metabolic health [93]. It is possible that the pair of “anti-inflammatory” and “pro-metabolic” medications has the potential to both snuff out the “fire of inflammation” and also “restart the cell’s metabolic engine,” thereby more effectively severing the vicious cycle.

Targeting the gut microbiota is another promising avenue for systemic interventions. The homeostasis of the gut microbiota is closely interconnected with the immune and metabolic processes of the host, and its dysbiosis is an important source of chronic low-grade inflammation and metabolic disorders [94]. Impaired gut barrier function predisposes to the passage of endotoxins like lipopolysaccharide (LPS) into the blood, where it chronically stimulates a systemic inflammatory response through engagement of pattern recognition receptors like TLR4. Therefore, restoring the healthy gut microbiome through supplementing with specific probiotics/prebiotics, or “format-rebooting” the entire microbiota through fecal microbiota transplantation (FMT), can be a potent, non-surgical method for alleviating the inflammatory burden at source and improving insulin resistance, and indirectly benefit the muscle-bone health [95].

Furthermore, directly targeting the key determinants of the muscle-bone signaling network is also a feasible approach for precision intervention. Developing drugs that mimic the action of therapeutic myokines/osteokines (e.g., irisin) or monoclonal antibodies against detrimental factors (e.g., myostatin or sclerostin) yields additional possibilities for clinical treatment. In fact, the anti-sclerostin antibody (romosozumab) was approved to treat osteoporosis, and its strong bone-forming effect demonstrates the validity of targeting this network [96]. Down the line, the application of such drugs in conjunction with the aforementioned anti-inflammatory and pro-metabolic treatments holds the potential for even more comprehensive and specific control of the muscle-bone unit.

Finally, the pivotal role of lifestyle interventions must be emphasized. Regular exercise, and in particular a combination of resistance training and aerobic exercise, is now solidified as the most effective non-pharmacologic therapy [97]. From the model presented in this review, the dramatic effectiveness of exercise lies in its ability to break the vicious cycle on multiple fronts simultaneously: it not only provides the necessary mechanical stimulation through muscle contraction to generate muscle-bone anabolism but also stimulates muscles to secrete irisin and other myokines with dual anti-inflammatory and pro-metabolic effects [98]. In the meantime, it activates the AMPK pathway, increases mitochondrial biogenesis, and improves systemic insulin sensitivity [99]. Exercise is therefore a natural multi-target regulator of the “inflammation-energy metabolism axis.”

### Unanswered Questions and Future Research Directions

Although this integrated paradigm provides profound insights for understanding sarcopenia-osteoporosis, there are still some basic scientific questions to be answered, which also point the way for future research.

First and foremost is the question of “causality and temporality” in disease onset. In the development of sarcopenia-osteoporosis, which comes first—inflammation or deranged energy metabolism? Or are they, from the very beginning, inseparably linked in a cause-and-effect relationship? Clarification of their initiating and secondary roles at different stages of the disease plays a key role in establishing the optimum window for early intervention and the preferred therapeutic strategy.

Second, the establishment of a distinct “biomarker panel” that can reflect the condition of the “inflammation-metabolism axis” in a precise manner is a prerequisite for the realization of precision medicine. The single markers currently used in clinical practice (e.g., CRP or blood glucose) do not suffice to completely reflect the dynamic changes of this complex network. Future research needs to integrate genomics, proteomics, and metabolomics data to discern a composite biomarker profile that is capable of holistically reflecting inflammatory levels, mitochondrial function, insulin sensitivity, and senescent cell burden. This will provide a powerful tool for early diagnosis, risk stratification, and objective measurement of therapeutic efficacy [100].

Furthermore, the “tissue heterogeneity” of senescent cells and their unequal impact on the musculoskeletal system are also worthy of serious investigation. Senescent cells of different tissue types (e.g., immune, endothelial, or adipose cells) may possess distinctively different SASP profiles and functions. For example, senescent cells in adipose tissue may primarily facilitate metabolic dysregulation and lipotoxicity by secreting adipokines and pro-inflammatory cytokines, while senescent immune cells may directly cause systemic inflammation to a larger extent. Clarification of such heterogeneity will allow us to develop more targeted senolytic therapies to achieve “precision clearance.”

Finally, “precision-targeted drug delivery” represents an important technological bottleneck to avoid the side effects of existing therapies and increase efficacy. Many potentially useful drugs (e.g., broad-spectrum anti-inflammatory agents or metabolic regulators) may have systemic side effects, e.g., immunosuppression or interference with the normal metabolism of other organs. Future research must be focused on the development of novel drug delivery systems, e.g., by employing nanotechnology or antibody-drug conjugates (ADCs), to target therapeutic agents directly to diseased muscle, bone, or senescent adipose tissue. This would maximize local therapeutic impact with negligible systemic side effects, indeed achieving safe and effective therapy.

## Conclusion

The sarcopenia-osteoporosis comorbidity is based on a vicious cycle that is driven by chronic low-grade inflammation and deranged energy metabolism. The logic in this review is that the disruption of this “inflammation-energy metabolism axis” is the underlying pathophysiological connection systemically linking muscle atrophy and bone loss. With aging, cellular stress signals, as represented by the activation of NLRP3 inflammasome and overwhelming ROS, cause a chronic increase in pro-inflammatory cytokines such as TNF-α and IL-6. This pro-inflammatory environment not only directly causes muscle and bone cell destruction through mechanisms like NF-κB but, more critically, systemic dysregulated energy metabolism is initiated by inhibiting the insulin signaling pathway and mitochondrial function. This metabolic dysregulation, in turn, as evidenced by mitochondrial dysfunction and lipotoxicity, releases more DAMPs that further amplify the inflammatory response and thus lock the entire system in this self-sustaining, vicious cycle. Therefore, therapeutic approaches to sarcopenia-osteoporosis must shift from addressing one tissue or target in isolation to interfering with this key vicious cycle. Future research must be directed at defining and targeting nodal points of convergence in the inflammation and metabolism network, e.g., with medications possessing both anti-inflammatory and metabolism-stimulating characteristics. Furthermore, the development of novel strategies that can precisely regulate mitochondrial homeostasis, inhibit specific inflammasome activity, or remodel the gut microbiome is likely to provide an breakthrough in the radical treatment of this progressively debilitating geriatric comorbidity.

## Supplementary Information

Below is the link to the electronic supplementary material.

Supplementary Material 1

## Data Availability

All data generated or analysed during this study are included in this published article.
